# COVID-19 outbreaks in a transmission control scenario: challenges posed by social and leisure activities, and for workers in vulnerable conditions, Spain, early summer 2020

**DOI:** 10.2807/1560-7917.ES.2020.25.35.2001545

**Published:** 2020-09-03

**Authors:** The national COVID-19 outbreak monitoring group, National COVID-19 outbreak monitoring group, National COVID-19 outbreak monitoring group, Bernardo R Guzmán Herrador, Silvia Rivera Ariza, Pello Latasa Zamalloa, Andrea Chong Valbuena, Paula Julián Pachés, Susana Monge, Lucía García San Miguel, Oscar Pérez Olaso, Teresa Sánchez Sagrado, Myrian Pichiule Castañeda, Berta Suárez Rodríguez, María José Sierra Moros, Fernando Simón Soria, , Montserrat García Gómez, Carmen Varela Martínez, Concepción Delgado Sanz, Rocío Amillategui Dos Santos, Nicola Lorusso, Alberto Carmona Ubago, Enric Durán Pla, Cristina Fernández Jiménez, Miriam García Vázquez, Ana Delia Cebollada Gracia, An LD Boone, Ana Fernández Ibáñez, Blanca Álvarez Fernández, Jaume Giménez Durán, Mercedes Caffaro Rovira, Magdalena Salom Castell, Magdalena Lucía Rojo Moreno, Alicia Pla Francés, Luis Viloria Raymundo, Soraya Curiel-Olmo, Manuel Galán Cuesta, Mireia Jané, Ana Martínez, Conchita Izquierdo, Ana Isabel Rivas Pérez, Violeta Ramos Marín, Irene López Corrales, Remedios Rodolfo Saavedra, María José Ruíz Pérez, Sara García Hernández, María García López, María del Henar Marcos Rodríguez, Cristina Ruíz Sopeña, Olga Lucía Rodríguez Arévalo, Sonia Blasco Guillem, Ana Taberner Cuevas, María del Mar López-Tercero Torvisco, Cristina Andreu Salete, Belén Montaño González, Ana Isabel González Villar, Olaia Pérez Martínez, Isabel Losada Castillo, Inmaculada Rodero Garduño, Jesús Sánchez Diaz, Susana Jiménez Bueno, Daniel Castrillejo Pérez, Luisa F. Hermoso Castro, Atanasio A. Gómez Anés, Ana García-Fulgueiras, M. Dolores Chirlaque, M. Isabel Barranco, Manuel García Cenoz, Carmen Sayón-Orea, Itziar Casado, Madalen Oribe Amores, Olatz Mokoroa Carollo, Ana Carmen Ibáñez Pérez, Eva Martínez Ochoa, María Puy Martínez Zárate

**Affiliations:** 1 The members of national COVID-19 outbreak monitoring group are listed below

**Keywords:** outbreak, COVID-19, Spain, transmission

## Abstract

Severe acute respiratory syndrome coronavirus 2 community-wide transmission declined in Spain by early May 2020, being replaced by outbreaks and sporadic cases. From mid-June to 2 August, excluding single household outbreaks, 673 outbreaks were notified nationally, 551 active (>6,200 cases) at the time. More than half of these outbreaks and cases coincided with: (i) social (family/friends’ gatherings or leisure venues) and (ii) occupational (mainly involving workers in vulnerable conditions) settings. Control measures were accordingly applied.

In this study, outbreaks notified to the national level in Spain during early summer of 2020 are reported. Moreover, certain settings where outbreaks were most frequently identified are described, as well as national, regional and local efforts to further investigate and control the outbreaks.

## National strategy for early detection, surveillance and control of coronavirus disease

During the period from April to beginning of May 2020, community-wide transmission of severe acute respiratory syndrome coronavirus 2 (SARS-CoV-2), the virus causing coronavirus disease (COVID-19), considerably decreased in Spain. On 21 June, following a gradual relief of lockdown measures, the state of alarm that had been declared in the country on 14 March 2020 [1] due to the COVID-19 pandemic ended. At this point, all of the country’s autonomous communities (i.e. regions) had to focus on transmission control, which required adopting measures to prevent a resurgence of COVID-19 cases. In a situation where population mobility increased progressively, early detection, investigation, and control of SARS-CoV-2 were considered of paramount importance.

In this context, the Royal Decree-Law 21/2020, of 9 June, on urgent prevention, containment, and coordination measures to deal with the public health crisis caused by COVID-19, was approved [2]. Under this robust legal framework, the national strategy for early detection, surveillance and control of COVID-19, issued on 11 May was adjusted in mid-June [3] with three main objectives: (i) ensure early detection of all cases with active SARS-CoV-2 infection, (ii) establish timely control measures to avoid new infections, and (iii) make all the information needed for epidemiological surveillance available, and effectively coordinate all levels of response.

Local and regional public health authorities were responsible for COVID-19 outbreak detection, investigation, and control (including isolation of cases), early identification and quarantine of contacts, as well as implementation of control measures in settings where outbreaks were occurring. Under the framework of the national strategy, all regions were to notify at national level every identified COVID-19 outbreak, except those involving only members of a single household, at the time of detection.

## Outbreak definition and notification procedures

For notification purposes, an outbreak was defined as three or more cases with active SARS-CoV-2 infection (as described in [3]), among whom an epidemiological link was established. Outbreak notification to the national level was performed by email using a specific template including the following information: municipality, number of cases, date of symptom onset of the first and last cases (or date of diagnosis if unknown or asymptomatic), number of related contacts and transmission setting.

The setting classification was not predefined and it was intended to be dynamic and flexible, in order to characterise the outbreaks, as they were occurring, into different categories according to risk groups or measures that had to be taken. At the time of publication we had the following setting classification: healthcare facility, long-term-care facility, socially vulnerable populations such as migrants arriving to Spain on small boats (‘pateras’), or linked to facilities for asylum-seekers and refugees, occupational setting, social setting, outbreaks affecting families living in different households and mixed outbreaks, where transmission moved, for instance, from the family environment to other population groups or vice–versa. The setting classification included additional subcategories, to allow detailed analyses if needed. For example, under the category ‘occupational setting’, further characterisation as the fruit and vegetable sector, meat processing plants, where people worked etc. was possible.

Outbreaks were classified as active (new cases diagnosed within the last 14 days), open (new cases diagnosed within the last 28 days) and closed (no new cases in the last 28 days).

All notifications were compiled in a database and analysed daily at the Coordinating Centre for Health Alerts and Emergencies (CCAES) at the Ministry of Health. Every day, an internal report was distributed within the National Surveillance and Alert Network describing the demographic and epidemiological characteristics of the outbreaks. Twice a week, a summary of the outbreak situation report was made publicly available on the Ministry of Health’s website [4] and was presented in the technical press conference hosted at the Ministry of Health.

## Description of outbreaks detected in early summer 2020

For the purpose of this study, we also took into account four outbreaks that had been notified end of May 2020, just before the official adjustment of the national strategy. Including these, from approximately mid-June up to 2 August (end of week 31), a total of 673 COVID-19 outbreaks affecting more than 8,300 persons had been notified to the national level in Spain. Of these, 551 outbreaks with more than 6,200 cases, were active on 2 August. At that point, all but one Spanish region had notified outbreaks.

Around 76% of all reported outbreaks (516/673) were small outbreaks with 10 cases or less. Around 2% of the outbreaks (12/673) had more than 100 cases. There had been 282 hospital admissions reported linked to these outbreaks; a certain number of them were not due to clinical criteria, but to isolate patients for whom appropriate isolation at home could not be guaranteed. 

The number of new outbreaks notified each week exponentially increased from 12 outbreaks during week 24, to 70 in week 28, and 234 in week 31. However, not all outbreaks notified each week corresponded to outbreaks being detected on that specific week, as some outbreaks were notified at a later stage to national level due to information consolidation processes at the local and regional level. 

The analysis of outbreaks with date of onset of symptoms of the first case after 1 June showed that their numbers increased during June and mid-July (Figure). At least 22,800 close contacts had been or were being followed up by local public health authorities during the investigation of these outbreaks. This number was certainly underestimated as this information was not always well completed or updated.

**Figure:**
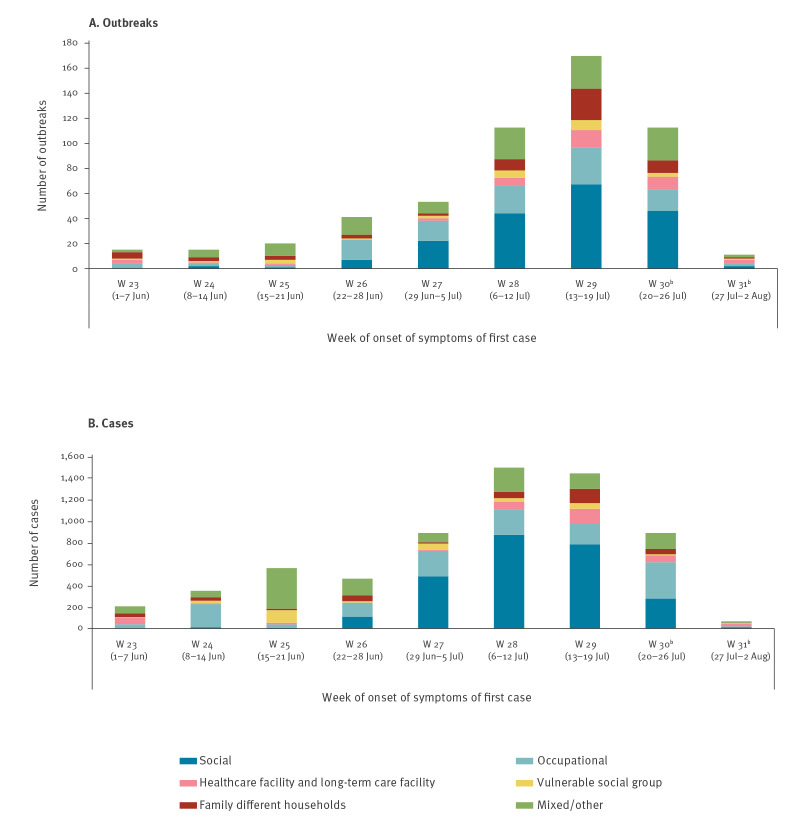
Number of COVID-19 (A) outbreaks and (B) cases by setting and week of onset of symptoms of first case, Spain, 1 June–2 August 2020 (n = 548 outbreaks)^a^

COVID-19: coronavirus disease; W: week.

^a^ Of the total of 673 COVID-19 outbreaks notified until 2 August 2020, only outbreaks where the first case had a known symptom onset/diagnosis date after 1 June are included. Of the 673 outbreaks, 31 outbreaks had a first case in which onset of symptoms was before 1 June, 21 outbreaks had an asymptomatic first case and for 73 outbreaks the diagnosis/symptom onset of first case was not registered (21 in healthcare facilities and long-term-care facilities, seven in vulnerable social groups, four in families related to different households, 18 in occupational settings, nine in mixed settings, 13 in social settings and one in an ‘other’ setting).

^b^ Information on week 30 and week 31 may not yet be complete.

The panel B of the Figure reflects the same outbreaks as panel A, but describes the cases (numbers and related setting) involved these outbreaks.

## Outbreak settings

There were two main settings where over 55% of active outbreaks (303/551) and over 60% (3,815/6,208) of active outbreak cases originated (Table). First, social settings, which comprised 35% (193/551) of all active outbreaks. Among these, the most frequent ones were those related to family gatherings or private parties (112 outbreaks including 854 cases), followed by those linked to leisure venues such as bars, restaurants, or clubs, with fewer outbreaks (n = 34) but involving more than 1,230 cases. Outbreaks linked to leisure venues tended to generate a considerable burden in contact-tracing activities. Several regions conducted active case finding campaigns for contact identification in these settings through calls in mass and social media or organising walk-through testing campaigns for target populations. We observed a large increase of outbreaks linked to social settings during the weeks included in the study period (Figure).

**Table:** Total and active notified COVID-19 outbreaks and number of cases by setting Spain, 29 May^a^–2nd August 2020 (n = 673 outbreaks)

Setting		Total				Active			
		Oubreaks		Cases		Oubreaks		Cases	
		N	%	N	%	N	%	N	%
Healthcare facility		20	3.0	274	3.3	17	3.1	219	3.5
Long-term care facility		59	8.8	829	9.9	39	7.1	376	6.1
Vulnerable social group		44	6.5	576	6.9	32	5.8	337	5.4
Family- different households		65	9.7	406	4.8	52	9.4	315	5.1
Occupational	Total	146	21.7	2,331	27.8	110	20.0	1,269	20.4
	Slaughterhouse/meat plant	19	NA	767	NA	12	NA	365	NA
	Agriculture seasonal worker/fruit-vegetable company	45	NA	1,022	NA	31	NA	500	NA
	Other/not specified	82	NA	542	NA	67	NA	404	NA
Social	Total	206	30.6	2,627	31.3	193	35.0	2,546	41.0
	Organised event/public space	31	NA	349	NA	29	NA	324	NA
	Family/friends reunion or private party	120	NA	900	NA	112	NA	854	NA
	Leisure facility (restaurant, bar, club…)	35	NA	1,234	NA	34	NA	1,231	NA
	Other/not specified	20	NA	144	NA	18	NA	137	NA
Mixed		111	16.5	1,218	14.5	92	16.7	1,050	16.9
Other		22	3.3	129	1.5	16	2.9	96	1.5
Total		673	100	8,390	100	551	100	6,208	100

NA: not applicable.

^a^ While most outbreaks (n=669) occurred from mid-June, four outbreaks, which were notified during the last days of May before the official adjustment of the national strategy, are included in the overview.

The second group of outbreaks was linked to occupational settings, representing 20% of all active outbreaks (110/551). Among these, outbreaks related to workers in the fruit and vegetable sector in conditions making them potentially vulnerable to COVID-19 were the most frequent, with 31 identified active outbreaks and around 500 cases. Workers at slaughterhouses or meat processing plants were the second most affected group, with at least 12 outbreaks and around 360 cases identified. The number of new outbreaks in this setting remained relatively stable throughout the study period. (Figure).

Additionally, around 17% of all active outbreaks had a mixed setting. Although less frequent, other relevant outbreaks were related to long-term care facilities (7%) and healthcare facilities (3%), and those involving other socially vulnerable groups (6%). Finally, outbreaks that affected multiple family households where a specific social event had not been identified, accounted for 9% of all outbreaks. The real proportion of outbreaks in family environments was certainly larger, keeping in mind that outbreaks involving one single household were not notifiable to the national level.

## Follow-up and control measures

The analysis of outbreak profiles in Spain, after lockdown measures were relieved, shows two main settings to target efforts.

Social gatherings involving families and/or friends and leisure settings, where suboptimal or lack of adherence to prevention and physical distancing measures constituted a major risk factor, likely due to a low risk perception. Awareness campaigns focused on these groups were carried out, with special emphasis on young people and night and social life. In addition, several regions implemented strict measures such as limiting capacity and opening hours for these venues, closing dance floors, or even closing indoors leisure venues [5,6].

Outbreaks among workers in vulnerable situations, were addressed with coordinated actions at national and regional level. Authorities from both sectors, health and agriculture, and stakeholders worked to ensure compliance of the occupational safety and labour regulations [7] and improve workers’ social and living conditions. This included organisational aspects such as establishing fixed groups of workers and reducing the number of workers per shift, guaranteeing physical distancing during transport and access to the working places, ensuring access to sick leave and isolation and quarantine facilities if needed, and increasing inspections to guarantee the application of these measures and regulations [8,9].

## Discussion and conclusions

After the relief of lockdown measures in Spain, the number of COVID-19 outbreaks detected increased rapidly during the early summer. This shows in part the great efforts that local and regional authorities are doing in early detection of cases and contact tracing, with much higher case detection capacity compared with the phase of wide community transmission. However, it also shows that the increase in mobility is associated to an increase in the circulation of the virus in Spain. New cases and cumulative incidence are currently increasing in almost all regions, including large outbreaks ending up, to some degree, as local community transmission in certain areas. A national plan for early response was approved by the Spanish health authorities to reduce the impact of the pandemic by strengthening rapid, timely and effective interventions and capacities to face an increase in transmission [10]. Efforts should continue to maintain and sustain the transmission control situation. The characterisation of outbreaks is essential to identify groups at greatest risk and to establish early measures aimed at controlling transmission, not only in the areas where they are occurring, but also preventively in the rest of the country.

## National COVID-19 outbreak monitoring group affiliations

**Coordinating Centre for Health Alerts and Emergencies (CCAES), Directorate General of Public Health, Ministry of Health, Madrid, Spain:** Bernardo R. Guzmán Herrador, Silvia Rivera Ariza, Pello Latasa Zamalloa, Andrea Chong Valbuena, Paula Julián Pachés, Susana Monge, Lucía García San Miguel, Oscar Pérez Olaso, Teresa Sánchez Sagrado, Myrian Pichiule Castañeda, Berta Suárez Rodríguez, María José Sierra Moros, Fernando Simón Soria (second affiliation: CIBER Epidemiologia y Salud Pública, CIBERESP). **Environmental and Occupational Health Unit, Directorate General of Public Health, Ministry of Health, Madrid, Spain:** Montserrat García Gómez. **National Centre of Epidemiology, Carlos III Health Institute:** Carmen Varela Martínez (second affiliation: CIBER Epidemiologia y Salud Pública, CIBERESP), Concepción Delgado Sanz (second affiliation: CIBER Epidemiologia y Salud Pública, CIBERESP), Rocio Amillategui Dos Santos. **Andalucía:** Dirección General de Salud Pública y Ordenación Farmacéutica, Consejería de Salud y Familias. Junta de Andalucía, Spain: Nicola Lorusso, Alberto Carmona Ubago, Enric Durán Pla. **Aragón:** Servicio de Vigilancia en Salud Pública, Dirección General de Salud Pública de Aragón, Spain: Cristina Fernández Jiménez, Miriam García Vázquez, Ana Delia Cebollada Gracia. **Asturias:** Servicio de Vigilancia Epidemiológica, Salud Pública, Asturias, Spain: An LD Boone, Ana Fernández Ibáñez, Blanca Álvarez Fernández. **Islas Baleares:** Servicio de Epidemiología, Dirección General Salud Publica, Islas Baleares, Spain: Jaume Giménez Durán, Mercedes Caffaro Rovira, Magdalena Salom Castell. **Canarias:** Servicio de Epidemiología y Prevención Dirección General de Salud Pública, Servicio Canario de la Salud, Spain: Magdalena Lucía Rojo Moreno, Alicia Pla Francés. **Cantabria.** Dirección General de Salud Pública, Cantabria, Spain: Luis Viloria Raymundo, Soraya Curiel-Olmo, Manuel Galán Cuesta. **Cataluña:** Subdirección General de Vigilancia y respuesta a Emergencias de Salud Pública, Agencia de Salud Pública de Catalunya, Spain: Mireia Jané, Ana Martínez, Conchita Izquierdo. **Ceuta:** Servicio de Vigilancia Epidemiológica, Consejería de sanidad, Consumo y Gobernación, Ciudad Autónoma de Ceuta, Spain: Ana Isabel Rivas Pérez, Violeta Ramos Marín, Irene López Corrales. **Castilla la Mancha:** Remedios Rodolfo Saavedra (Servicio de Epidemiología de Castilla-La Mancha), María José Ruíz Pérez (Sección de Epidemiología, Delegación Provincial de Sanidad de Toledo), Sara García Hernández (Servicio de Epidemiología de Castilla-La Mancha). **Castilla y León**: Servicio de Epidemiología, Dirección General de Salud Pública, Consejería de Sanidad, Junta de Castilla y León, Spain: María García López, María del Henar Marcos Rodríguez, Cristina Ruíz Sopeña. **Comunidad Valenciana**: Subdirección General de Epidemiología, Vigilancia de la Salud y Sanidad Ambiental, Conselleria de Sanitat Universal i Salut Pública de la Comunitat Valenciana. Spain: Olga Lucía Rodríguez Arévalo (second affiliation: PhD student Universidad Politécnica de Valencia), Sonia Blasco Guillem, Ana Taberner Cuevas. **Extremadura:** Subdirección de Epidemiología, Dirección General de Salud Pública, Servicio Extremeño de Salud, Consejería de Sanidad y Políticas Sociales, Junta de Extremadura, Spain: María del Mar López-Tercero Torvisco, Cristina Andreu Salete, Belén Montaño González. **Galicia**: Servizo de Epidemioloxía Dirección Xeral de Saúde Pública, Consellería de Sanidade Xunta de Galicia, Spain: Ana Isabel González Villar, Olaia Pérez Martínez, Isabel Losada Castillo. **Madrid:** Subdirección General de Epidemiología de la Comunidad de Madrid, Spain: Inmaculada Rodero Garduño, Jesús Sánchez Diaz, Susana Jiménez Bueno. **Melilla:** Servicio de Epidemiología, Dirección General de Salud Pública y Consumo, Melilla, Spain: Daniel Castrillejo Pérez, Luisa F. Hermoso Castro, Atanasio A. Gómez Anés. **Murcia:** Department of Epidemiology, Murcia Regional Health Council, IMIB-Arrixaca, Murcia, Spain. Epidemiology and Public Health Network of Biomedical Research Centre, CIBERESP, Spain: Ana García-Fulgueiras, M. Dolores Chirlaque, M.Isabel Barranco. **Navarra:** Servicio de Epidemiología y Prevención Sanitaria. Instituto de Salud Pública de Navarra, IdiSNA: Manuel García Cenoz, Carmen Sayón-Orea, Itziar Casado (second affiliation: CIBER Epidemiologia y Salud Pública, CIBERESP). **País Vasco:** Madalen Oribe Amores (Unidad de Epidemiologia, Subdirección de Salud Pública y Adicciones de Bizkaia, Spain), Olatz Mokoroa Carollo (Unidad de Epidemiologia, Subdirección de Salud Pública de Gipuzkoa y Adicciones, IIS Biodonostia, Spain). **La Rioja:** Servicio de Epidemiología y Prevención Sanitaria. Dirección General de Salud Pública, Consumo y Cuidados. Consejería de Salud de La Rioja, Spain: Ana Carmen Ibáñez Pérez, Eva Martínez Ochoa, María Puy Martínez Zárate.
